# The Contribution of Childhood Parental Rejection and Early Androgen Exposure to Impairments in Socio-Cognitive Skills in Intimate Partner Violence Perpetrators with High Alcohol Consumption

**DOI:** 10.3390/ijerph10083753

**Published:** 2013-08-20

**Authors:** Ángel Romero-Martínez, Marisol Lila, Alba Catalá-Miñana, Ryan K. Williams, Luis Moya-Albiol

**Affiliations:** 1 Department of Psychobiology, University of Valencia, Valencia 46010, Spain; E-Mail: angel.romero@uv.es; 2 Department of Social Psychology, University of Valencia, Valencia 46010, Spain; E-Mails: marisol.lila@uv.es (M.L.); alba.catala@uv.es (A.C.-M.); 3 Criminal Justice Department, University of Illinois Springfield, Springfield, IL 62703, USA; E-Mail: rwill2@uis.edu

**Keywords:** alcohol, childhood abuse, intimate partner violence, socio-cognitive skills, 2D:4D ratio

## Abstract

Alcohol consumption, a larger history of childhood parental rejection, and high prenatal androgen exposure have been linked with facilitation and high risk of recidivism in intimate partner violence (IPV) perpetrators. Participants were distributed into two groups according to their alcohol consumption scores as high (HA) and low (LA). HA presented a higher history of childhood parental rejection, prenatal masculinization (smaller 2D:4D ratio), and violence-related scores than LA IPV perpetrators. Nonetheless, the former showed poor socio-cognitive skills performance (cognitive flexibility, emotional recognition and cognitive empathy). Particularly in HA IPV perpetrators, the history of childhood parental rejection was associated with high hostile sexism and low cognitive empathy. Moreover, a masculinized 2D:4D ratio was associated with high anger expression and low cognitive empathy. Parental rejection during childhood and early androgen exposure are relevant factors for the development of violence and the lack of adequate empathy in adulthood. Furthermore, alcohol abuse plays a key role in the development of socio-cognitive impairments and in the proneness to violence and its recidivism. These findings contribute to new coadjutant violence intervention programs, focused on the rehabilitation of basic executive functions and emotional decoding processes and on the treatment of alcohol dependence.

## 1. Introduction

Despite the fact that not all men involved in intimate partner violence (IPV) abuse alcohol or other drugs, a large percentage of them attack their partners under the effects of these substances [1]. Alcohol consumption has been linked to the facilitation of IPV [2,3] and to high risk of recidivism or maintenance of this type of violence [4]. It may also produce a negative reinforcement that mitigates negative emotions and so entails the perpetuation of violence [5]. Moreover, recent research has revealed that highly violent IPV perpetrators presented higher alcohol abuse or dependence than those with lower levels of violence [6].

The current model for explaining this phenomenon is referred to as the *Myopic Model* [7], and states that alcoholic intoxication worsens attention capacities and/or information processing. Therefore, the quantity of stimuli one person is able to process decreases and this, in turn, facilitates a violent reaction [8]. Furthermore, chronic alcohol abuse affects many cognitive skills. In this sense, alcohol abuse has been related to poor academic performance explained by deficits in working memory and attention and verbal learning [9,10], decision making [11], verbal skills [12], as well as long and short term memories [13]. These impairments extend to socio-cognitive skills as well. Thus, alcohol dependents presented diminished emotional decoding abilities [14,15], theory of mind deficits and humor processing difficulties [16]. Of all of these effects, the most extensively studied deficits have been the executive function and memory, mainly due to their greater vulnerability to the toxic effects of alcohol. Moreover, alcohol-related aggression was stronger in healthy social drinker men with lower cognitive and emotional empathy [17] and high hostile sexism [18]. Therefore, chronic alcohol consumption deficits exacerbate deficits already present in substance abusers. Part of those deficits could be explained by the side effects of childhood abuse and/or rejection, which in turn, increases the likelihood to adopt risky behaviors such as alcohol or drug abuse during adolescence and young adulthood [19].

Suffering maltreatment during childhood is an important factor for the intergenerational transmission of violence [20]. Nonetheless, maltreatment with traumatic brain injury is not enough to explain the diminished socio-cognitive skills in IPV perpetrators [21].

On the other hand, the 2D:4D ratio, a peripheral marker of prenatal masculinization of the Central Nervous System (CNS), may be considered as an indicator of the adoption of reckless behaviour, such as alcohol or other drug abuse [22,23]. It has been linked to male sensation seeking [24], physical aggression [25], left-handedness [26], attention-deficit-hyperactivity disorder [27] and a genetic polymorphism of the androgen receptor [28]. All this aspects were related, in turn, to vulnerability for alcohol dependency. Hence, the 2D:4D ratio of the right hand of males could be used as trait marker in identifying patients with alcohol dependency [22]. Nevertheless a lack of relationship between alcohol drinking and 2D:4D ratio in healthy young men has also been described [29].

With all this in mind, the main goal of this study was to understand the specific cognitive differences among IPV perpetrators with high and low alcohol abuse, using several cognitive-affective and neuropsychological parameters and measures of violence-related attitudes and beliefs and prenatal masculinization. The relationship between alcohol abuse, socio-cognitive impairments, and high impulsivity and anger traits may prone to physical and psychological domestic violence as well as risk of violence recidivism after treatment [20,21,22,23,24,25,26,27,28,29,30,31,32]. For this reason, we hypothesized that HA abuse IPV perpetrators would have greater deficits in cognitive flexibility, cognitive empathy and emotional decoding abilities, and greater impulsivity, anger temperament and expression and risk of recidivism when compared with LA IPV perpetrators. In addition, a history of childhood parental rejection could increase the presence of alcohol and other drugs abuse during adulthood [20]. Thus, we expected to find that IPV perpetrators with high alcohol abuse would present higher rates of history of childhood parental rejection. Furthermore, a high rate of history of childhood parental rejection would be associated to low cognitive flexibility which could explain the persistence of sexist stereotypes that frequently appear in IPV perpetrators [33]. Moreover, parental rejection would be related to poor emotional decoding abilities (which constitute an initial stage of social information processing) and it would produce deficits in the “theory of mind” (ToM) and, consequently, in how understanding a partner’s perspective or feelings [20,33], especially in IPV perpetrators with high alcohol abuse. Finally, early testosterone exposure has been related to high impulsivity, which in turn may increase the proneness to behave violently after alcohol abuse [24]. Moreover, high prenatal CNS masculinization has been related to alcohol consumption [22], a fact that may cause impairments in several empathic skills as the emotional decoding abilities [14,15,16]. Hence, we would expect that a masculinized 2D:4D ratio would be associated to high impulsivity and poor emotional decoding process, which may increase the risk to behave violently. The analysis of these variables and their relationships may offer a wider explanation of the role of alcohol abuse in the relationship between neuropsychological deficits and the facilitation and maintenance of IPV. Our findings may also permit to delimitate the treatment for specific IPV perpetrators and contribute to the development of neuropsychological enrichment programs coadjutant to the psychotherapeutic and communitarian therapies.

## 2. Method

### 2.1. Participants

The final sample was composed of 145 IPV perpetrators volunteers who were recruited from the participants in the CONTEXTO psycho-educational and community-based treatment programme (mandatory for male abusers) at the Department of Social Psychology, University of Valencia. They had been sentenced to less than two years in prison and had no previous criminal record, and so benefitted from a sentence suspension subject to their attendance to an intervention programme [34]. The experiment was performed in accordance with the Helsinki Declaration and approved by the University of Valencia Ethics Committee.

### 2.2. Procedure

Each subject participated in three sessions in the psychology laboratories of the University of Valencia. In the first session participants were interviewed in order to identify (and subsequently reject) participants who suffered from organic or psychological diseases. The inclusion criteria were having no organic or mental illnesses. Moreover, IPV perpetrators were selected with similar anthropometrical and demographic characteristics. In the second session, after arriving at the laboratory, participants were taken to a room where they signed an informed consent to participate in the study, and demographic and anthropometrical variables (age and 2D:4D digit ratio) and alcohol consumption (AUDIT, CAGE and MILLON-III alcohol abuse) were registered. The session took place one day after the first session between 10 a.m. and 2 p.m. in order to avoid a fatigue effect due to the working day. In this session, two neuropsychological tests were administrated, the Wisconsin Card Sorting Test (WCST) and the reading the mind in the eyes (Eyes Test). Finally, participants in a third session completed a battery of questionnaires for evaluating violence-related attitudes and beliefs.

### 2.3. Alcohol Abuse Evaluation

The Spanish version of the Alcohol Use Disorders Identification Test (AUDIT) [35] incorporates questions about the quantity and frequency of alcohol use in adults. It has been developed by the World Health Organization (WHO) to identify persons whose alcohol consumption has become hazardous or harmful to health. It is composed of 10 self-report items ranging from 0 (never) to 4 (daily or almost daily). The AUDIT is distinguished from other well-known screens in the fact that items are scored on a frequency continuum (rather than dichotomously), it requests measures over a limited time period (e.g., 6 months *vs*. lifetime), and it appears to have broader applicability by discriminating hazardous and harmful drinkers (*i.e.*, at-risk problem drinkers) rather than those who are alcohol dependent [36]. The Cronbach alpha was 0.88.

We employed the Spanish adaptation of the *CAGE Test* [37]. This is a scale used to identify alcohol abuse. It includes four questions with dichotomous responses related to individual culpability, social criticism, the need to reduce consumption and morning ingestion. Each affirmative answer is given one point. Scores three or four suggest alcohol abuse, however, a participant with a score of two points or more is considered to potentially have problems with alcohol. For our sample, the internal consistency was 0.76 [38].

We employed the Spanish version of the *Millon Clinical Multiaxial Inventory-III* (*MCMI-III*) [39]. Self-report inventory consisting of 175 dichotomous items which measure personality disorders. It comprises three Modifying scales; 11 Clinical Personality Patterns scales; three Severe Personality scales, seven Clinical Syndromes scales, and three Severe Syndrome scales. The Spanish version validation reported reliability between 0.65 and 0.92. For this study, the Alcohol Dependence scales were used [40].

### 2.4. Psychological Trait Profiles

The Interpersonal Reactivity Index (IRI) assesses four aspects of empathic response [41]. We used the Spanish adaptation [42], which includes the four following subscales: perspective taking or tendency to take the psychological point of view of others;, fantasy or tendency to get caught up in fictional stories and imagine oneself in the same situations as fictional characters;, empathic concern orsympathy and concern for others; and personal distress or kinds of feelings (anxiety, *etc*.) that get in the way of helping others. It is ranked in a 5-point Likert scale with reliability coefficients ranging from 0.56 to 0.70.

Anger and its expression were measured by an adapted version [43] of the *State-Trait Anger Expression Inventory-2* (*STAXI-2*) [44]. This test is distributed into six subscales: two for evaluating trait anger (temperament and reaction) and four for anger expression (anger expression out, anger expression in, anger control out, and anger control in). To reduce the number of tests, increase power for effect size, and aid interpretation within a conceptual framework, trait anger subscales were combined into a single variable (T-Ang). Moreover, a general anger expression index (AEI) was calculated by adding the scores of the two expression subscales and subtracting the scores of the two control scales, and finally adding 36 units to avoid negative scores. The Cronbach’s alpha ranged from 0.67 to 0.89.

Impulsivity was measured by the Spanish version of the Plutchik Impulsivity Scale [45]. This 15-item scale measures impulsivity as an immediate reaction disregarding any behaviour consequences. It is a Likert-type scale with a 4-point response (1 = never; 4 = almost always) [46]. The Cronbach alpha was 0.72.

### 2.5. Neuropsychological Measures

The revised version of reading the mind in the eyes (Eyes Test) was administered. This task is considered an advanced theory of mind test which differently from the IRI (previously described) measures the emotional decoding process that contains 36 black and white photographs of the eye region of the face of different actors and actresses. Subjects must attribute the mental state of the actors. Participants were instructed to choose which of four words best described what the person in the photo was thinking or feeling. Scores are calculated as the total number of correct choices for all 36 photographs [47].

The Spanish revised version of the Wisconsin card sorting test (WCST) [48] was used to measure cognitive flexibility. Cards must be sorted until six categories are matched or until all 128 cards are sorted. Cards are matched according to different criteria such as colour, form, and number. After 10 consecutive correct cards are sorted, a new criterion is instituted without warning [49].

### 2.6. Violence-Related Attitudes and Beliefs Scales

Violence-related attitudes and beliefs scales were evaluated by the Spanish version of the Spousal Assault Risk Assessment Guide (SARA) [50]. The protocol was completed by trained researchers [34]. It includes a set of 20 risk factors for spousal violence. The risk factors are related to violence risk in general and risk of spousal violence specifically. Evaluators have to code the presence of each risk factor, whether any of the risk factors is considered “critical”, and the overall degree of risk posed by the participant. For this study, the sum of the risk factors was taken into account [51].

The Spanish version of *The Revised Conflict Tactics Scale* (*CTS2*) [52]. This is a self-report inventory to assess how individuals choose to resolve relationship conflicts. Participants report on the behaviors of themselves and their partners during conflict. The measure consists of 78 items 8-point Likert-type, where 0 means “This has never happened” and 6 means “More than 20 times in the past year”; however, 7 means “Not in the past year, but it happened before”. Cronbach alpha for the present study ranged from 0.78 to 0.84 for negotiation, physical assault and psychological aggression [53].

The Spanish version of the Ambivalent Sexism Inventory (ASI) [54]. This inventory is a 22-item self-report with two scales: Hostile and benevolent sexism. Participants indicate their level of agreement using a 6-point Likert-type (0 = strongly disagree; 5 = strongly agree) with high scores showing more sexist attitudes. Internal consistency for this sample was 0.89 (Hostile sexism) and 0.84 (Benevolent sexism) [55].

Parental Acceptance-Rejection Questionnaire (PARQ) [56]. Self-report questionnaire that measures participants’ perceptions of the way they were treated by their mothers and fathers. This instrument has been also widely used with Spanish samples [57]. It is composed of four factors: Parental Warmth and Affection, Parental Hostility and Aggression, Parental Indifference and Neglect, and Parental Undifferentiated Rejection. A total score was obtained by adding the scores of the four subscales and a high score showing high perceived rejection [56,57]. Cronbach alphas for this study were between 0.78 and 0.95.

### 2.7. 2D:4D Digit Ratio

The 2D:4D ratio was obtained by taking photocopies of the palms and fingers of both hands and measuring the length of the second to the fourth digit from the most proximal wrinkle in each finger to the tip—using a calliper for this purpose [33]. The ratio was calculated by dividing the length of the second by the fourth digit [58].

### 2.8. Data Analysis

Cluster analysis includes a variety of multivariate statistical procedures used to classify individuals into relatively homogeneous groups [59]. K-means cluster analyses were conducted to determine the subgroups. The cluster analyses focused on the following measures: (a) the AUDIT score (b) the CAGE score (c) the men’s scores on an alcohol abuse scale derived from the MCMI-III. The cluster analyses resulted in the formation of two groups. Seventy-four IPV perpetrators were grouped in the High Alcohol (HA), and 71 IPV perpetrators were placed in the Low Alcohol (LA) group.

It was previously established using the Kolmogorov-Smirnov statistic (*p* < 0.05) that the data was normally distributed. T-tests were conducted to test differences in all variables between HA and LA. Effect sizes for the between-group differences were calculated using Cohen’s d [60]. Chi square analyses were performed for demographic variables.

We used linear regression models to investigate whether the history of childhood rejection and 2D:4D ratio predicted violence and empathy indicators based on the theoretical models proposed in the introduction section. As recommend by Preacher, Rucker, & Hayes [61], we confirmed the association between the mediation variables, the independent and the dependent variables. Bootstrapping statistic (a powerful and accurate method) was employed to test the mediation models.

All statistical analyses were performed with SPSS 17.0 for Windows with the alpha level fixed at 0.05.

## 3. Results

Descriptive characteristics, psychological trait profiles and neuropsychological variables for the IPV perpetrators with HA and LA are presented in Table 1. There were significant differences between groups in marital status (χ²(1) = 16.85, *p* < 0.05), with more cases of divorced in HA than in LA IPV perpetrators. Moreover, the former showed a lower right-hand 2D:4D digit ratio (*t*(143) = −2.89, *p* = 0.004, *d* = 0.49).

**Table 1:** Mean ± SEM of descriptive characteristics, psychological trait profiles and neuropsychological variables for High Alcohol (HA) and Low Alcohol (LA) IPV perpetrators. * *p* < 0.05; ** *p* < 0.01.

		High Alcohol (*n* = 74)	Low Alcohol (*n* = 71)
Age (years)		38.34 ± 10.47	41.67 ± 11.21
Right 2D:4D ratio *		0.95 ± 0.09	0.99 ± 0.04
Left 2D:4D ratio		1.00 ± 0.05	0.99 ± 0.07
Educational level	Basics	43 (58%)	41 (58%)
	Graduate	25 (34%)	23 (32%)
	College	6 (8%)	7 (10%)
Nationality	Spanish	57 (77%)	55 (77%)
	Latin Americans	9 (12%)	6 (8.5%)
	Africans	6 (8%)	4 (6%)
	Russians	2 (3%)	6 (8.5%)
Employment status	Working full or part time	37 (50%)	39 (55%)
	Unemployed	37 (50%)	32 (45%)
Economic income per year	<1,800 €	17 (23%)	14 (20%)
	1,800–12,000 €	40 (54%)	33 (46%)
	12,000–36,000 €	17 (23%)	20 (28%)
	>36,000 €	0 (0%)	4 (6%)
Marital status *	Single	20 (27%)	18 (25%)
	Married	19 (26%)	34 (48%)
	Divorced	35 (47%)	19 (27%)
IRI perspective taking *		18.48 ± 7.41	23.14 ± 5.43
IRI empathic concern		24.21 ± 0.71	23.05 ± 0.61
IRI personal distress **		16.00 ± 0.93	12.50 ± 0.70
IRI fantasy		17.58 ± 0.92	18.35 ± 0.82
STAXI-2 T-Ang *		18.23 ± 5.24	14.13 ± 3.67
STAXI-2 AEI *		29.14 ± 10.96	22.70 ± 12.30
Plutchick (impulsivity) *		30.90 ± 6.49	25.28 ± 4.46
Eyes test *		21.5 ± 1.37	24.89 ± 0.88
Eyes test (positive emotions)		4.67 ± 0.36	5.11 ± 0.45
Eyes test (negative emotions)		7.50 ± 0.82	8.63 ± 0.39
Eyes test (neutral emotions) *		9.33 ± 0.60	11.16 ± 0.51
WCST total trials **		118.28 ± 4.45	100.68 ± 4.08
WCST total mistakes **		48.00 ± 5.24	17.89 ± 2.15
WCST perseverative mistakes **		13.78 ± 4.37	1.95 ± 0.36
WCST non perseverative mistakes **		34.16 ± 4.24	16.26 ± 2.03
WCST perseverative mistakes (%) **		16.22 ± 5.79	2.05 ± 0.47
WCST failure to maintain set		0.97 + 0.34	0.79 + 0.34
WCST trials to complete the first category		14.57 + 1.81	15.05 + 2.17
WCST number of categories **		3.56 + 0.47	4.00 + 0.55
WCST conceptual level		7.50 + 0.77	6.89 + 0.48
WCST learn to learn		3.22 + 0.56	6.00 + 0.00

### 3.1. Psychological Trait Profiles

Regarding IRI, HA IPV perpetrators showed lower scores in IRI perspective taking and higher scores in IRI personal distress than did LA participants (*t*(143) = −4.29, *p* = 0.000, *d* = 0.72) and (*t*(143) = 5.18, *p* = 0.000, *d* = 0.87), respectively. Regarding the STAXI-2, the former presented higher trait anger (*t*(142) = 5.36, *p* = 0.000, *d* = 0.90), AEI (*t*(143) = 3.29, *p* = 0.000, *d* = 0.55). Finally, the HA group presented higher impulsivity scores in the Plutchik questionnaire than the LA group (*t*(143) = 5.96, *p* = 0.000, *d* = 1.00).

### 3.2. Neuropsychological Variables

Data from the Eyes Test and the WCST are presented in Table 1. HA obtained a worse performance in the Eyes Test than LA IPV perpetrators (*t*(143) = −3.05, *p* = 0.003, *d =* 0.51). When the Eyes Test was classified depending on the emotional meaning and then including positive, negative and neutral emotions, the former obtained lower scores only in the case of neutral emotions (*t*(143) = −2.35, *p* = 0.02, *d* = 0.39).

Regarding the performance in the WCST, HA completed fewer number of categories (*t*(143) = −2.05, *p* = 0.042, *d* = 0.34) and committed more total (*t*(143) = 2.16, *p* = 0.032, *d* = 0.36), perseverative (*t*(143) = 2.33, *p* = 0.021, *d* = 0.39), percentage of perseverative (*t*(143) = 2.46, *p* = 0.015, *d* = 0.41) and non-perseverative errors (*t*(143) = 2.26, *p* = 0.025, *d* = 0.38) than LA IPV perpetrators. Additionally, the former used more trials to complete the categories (*t*(143) = 2.75, *p* = 0.007, *d* = 0.46). 

### 3.3. Violence-Related Attitudes and Beliefs Scales

HA IPV perpetrators presented higher scores in the SARA (which measure the risk of recidivism of violence (*t*(143) = 3.16, *p* = 0.002, *d* = 0.31), ASI hostile sexism (*t*(143) = 3.21, *p* = 0.002, *d* = 0.54) and ASI benevolent sexism scores (*t*(143) = 1.88, *p* = 0.062, *d* = 0.31) than LA IPV perpetrators. Regardless of the CTS, HA IPV perpetrators reported to have higher scores in physical assault and psychological aggression (*t*(143) = 3.41, *p* = 0.001, *d* = 0.57 and *t*(143) = 2.76, *p* = 0.006, *d* = 0.46, respectively) than LA IPV perpetrators. Additionally, HA IPV perpetrators showed higher scores in their PARQ (which measure the history of parental rejection (*t*(143) = 1.93, *p* = 0.056, *d* = 0.33) than LA IPV perpetrators (Table 2).

**Table 2:** Mean ± SEM of SARA, CTS, ASI, and PARQ for High Alcohol (HA) and Low Alcohol (LA) IPV perpetrators. * *p* < 0.05.

		High Alcohol (*n* = 74)	Low Alcohol (*n* = 71)
The Spousal Assault Risk Assessment Guide *(SARA)*Total score *		11.44 ± 5.99	9.16 ± 5.15
**Conflict Tactics Scale CTS**	Physical Assault *	4.61 ± 8.82	1.49 ± 4.02
	Psychological Aggression *	18.45 ± 26.60	8.63 ± 18.98
	**Negotiation**	41.77 ± 42.60	32.31 ± 38.18
**Ambivalent Sexism Inventory ** **ASI**	**Hostile**	2.96 ± 1.17	2.27 ± 1.36
	**Benevolent**	3.29 ± 1.06	2.93 ± 1.26
Parental Acceptance-Rejection Questionnaire Child Version PARQ *		224.11 ± 39.85	215.45 ± 49.17

### 3.4. Does History of Childhood Parental Rejection Play a Key Role in Adult Violence and Empathy, Mainly in HA IPV Perpetrators? Are Any Cognitive Processes Mediators of This Association?

**Figure 1:**
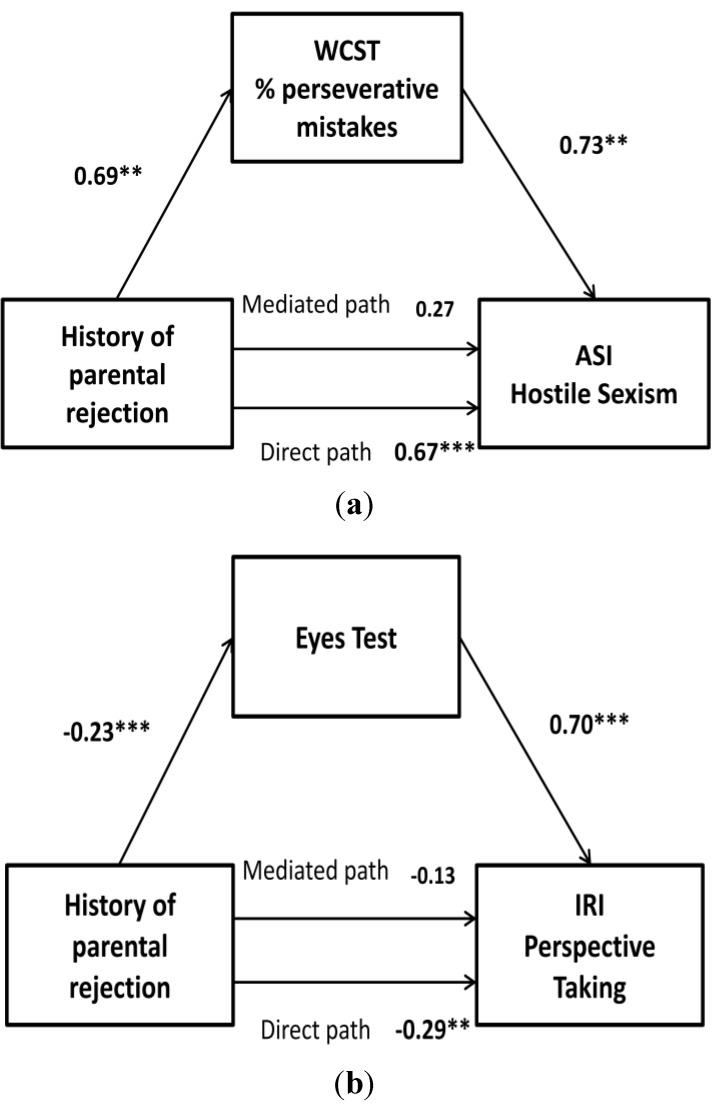
History of childhood parental rejection as a predictor of (**a**) ASI hostile sexism mediated by WCST% of perseverative mistakes and (**b**) IRI perspective taking mediated by eyes test in IPV perpetrators. ** *p* < 0.01; *** *p* < 0.001.

The direct effect of history of childhood parental rejection on WCST percentage of perseverative mistakes was statistically significant (β = 0.69, SE = 0.21, *p* < 0.001), as were the direct effects of WCST percentage of perseverative mistakes and of history of childhood parental rejection on their hostile sexism (β = 0.73, SE = 0.30, *p* < 0.001; and β = 0.67, SE = 0.15, *p* < 0.001, respectively). In addition, when accounting for the effect of sensitivity to WCST percentage of perseverative mistakes, the effect of history of childhood parental rejection on hostile sexism reduced to nonsignificance (β = 0.27, SE = 0.28, *p* > 0.05). Results of the bootstrap analysis indicate that mediation was present (M = 0.46, SE *=* 0.32; 95% CI = 0.08 to 1.84). Thus, it can be concluded that the association between history of childhood parental rejection and hostile sexism in HA IPV perpetrators was mediated by their high WCST percentage of perseverative mistakes (Figure 1(a)).

The direct effect of history of childhood parental rejection on eyes test was also statistically significant (β = −0.23, SE = 0.06, *p* < 0.001), as were the direct effects of history of childhood parental rejection on IRI perspective taking (β = 0.70, SE = 0.14, *p* < 0.001) and of eyes test on IRI perspective taking (β = −0.29, SE = 0.08, *p* < 0.001). Likewise, when accounting for the effect of sensitivity to eyes test, the effect of history of childhood parental rejection on their IRI perspective taking reduced to nonsignificance (β = −0.13, SE = 0.08, *p* < 0.05). Results of the bootstrap analysis indicate that mediation was present (*M* = 0.16, *SE* = 0.05; 95% CI = 0.07 to 0.28). Thus, the association between history of childhood parental rejection and IRI perspective taking in HA IPV perpetrators was mediated by their low eyes test (Figure 1(b)).

For analysing the relevance of alcohol consumption in increasing the probability of presenting higher violent behavior and lower empathy in adulthood, all analyses were performed separately for HA and LA IPV perpetrators. As expected, the regression models were significant in HA participants (for all *p* < 0.05), but not for LA IPV perpetrators.

### 3.5. Is Prenatal Masculinization Involved in Violence and Empathy during Adulthood, Mainly in HA IPV Perpetrators? Which Psychological or Cognitive Characteristics Mediate This Association?

**Figure 2:**
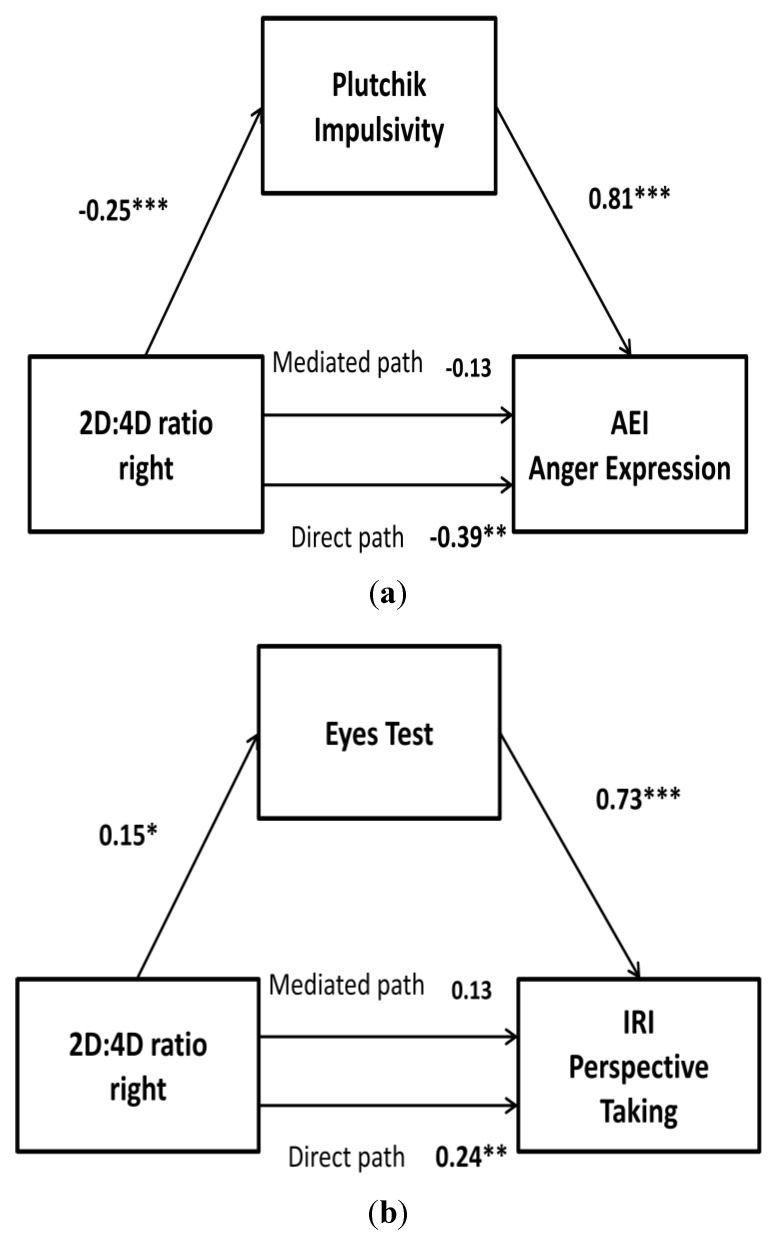
2D:4D ratio right as a predictor of (**a**) anger expression (AEI) mediated by impulsivity and (**b**) IRI perspective taking mediated by eyes test in IPV perpetrators. * *p* < 0.05; ** *p* < 0.01; *** *p* < 0.001.

The direct effect of 2D:4D ratio on Plutchik impulsivity was statistically significant (β = −0.25, SE = 0.07, *p* < 0.001), as were the direct effects of Plutchik impulsivity and of 2D:4D ratio on their AEI (β = 0.81, SE = 0.19, *p* < 0.001; and β = −0.39, SE = 0.13, *p* < 0.001, respectively). In addition, when accounting for the effect of sensitivity to Plutchik impulsivity, the effect of 2D:4D ratio on AEI reduced to nonsignificance (β = −0.13, SE = 0.13, *p* > 0.05). Results of the bootstrap analysis indicate that mediation was present (M = 0.21, SE = 0.09; 95% CI = 0.07 to 0.45). Thus, it can be concluded that the association between 2D:4D ratio and AEI in HA IPV perpetrators was mediated by their high Plutchik impulsivity (Figure 2(a)).

The direct effect of 2D:4D ratio on eyes test was statistically significant (β = 0.15, SE = 0.07, *p* < 0.05), as were the direct effects of eyes test and of 2D:4D ratio on their IRI perspective taking (β = 0.73, SE = 0.13, *p* < 0.000; and β = 0.24, SE = 0.09, *p* < 0.01, respectively). In addition, when accounting for the effect of sensitivity to eyes test, the effect of 2D:4D ratio on IRI perspective taking reduced to nonsignificance (β = 0.13, SE = 0.07, *p* > 0.05). Results of the bootstrap analysis indicate that mediation was present (*M* = 0.11, *SE* = 0.05; 95% CI = −0.02 to 0.25). Thus, it can be concluded that the association between 2D:4D ratio and IRI perspective taking in HA IPV perpetrators was mediated by their high eyes test (Figure 2(b)).

As previously indicated, all analyses were performed separately for HA and LA IPV perpetrators. Regression models were significant in HA participants (for all *p* < 0.05), but not for LA IPV perpetrators.

## 4. Discussion

HA IPV perpetrators presented lower 2D:4D ratio and cognitive empathy than LA IPV perpetrators. Nonetheless, the former showed higher personal distress, impulsivity, anger (expression and trait), risk of recidivism, violent conflict solutions tactics, sexism and history of childhood parental rejection than LA. With respect to neuropsychological and cognitive empathic features, HA IPV perpetrators reported to have poor executive performance and emotional recognition. Finally, particularly in HA IPV perpetrators, the history of childhood parental rejection was associated to high hostile sexism and low cognitive empathy. Moreover, the 2D:4D ratio was associated to high anger expression and low cognitive empathy. In both cases, a poor neuropsychological performance and high impulsivity mediated those relationships.

In our previous study with a small sample size, IPV perpetrators presented poor cognitive empathy and emotional decoding process than non-violent controls. However, personal distress was high in the former [33]. Our current data replicate these findings with a larger sample size. In line with these findings, emotional decoding processes are important for understanding thoughts and emotions and help to predict the behaviour of others [62]. Our data support that these deficits in emotional stimuli decoding process, especially in those with a neutral value, may explain why IPV perpetrators obtained worse scores in the perspective taking scale or cognitive empathy, particularly in those perpetrators with high alcohol consumption. Thus, poor scores in cognitive empathy could explain why IPV perpetrators misunderstand and attribute hostile connotations to neutral stimuli, increasing the likelihood of behaving aggressively [63]. That, in turn, contributes to an understanding as to why men commit domestic violence although women develop learned helplessness and why women did not show negative or positive emotions. Nonetheless, HA IPV perpetrators lacked cognitive, but not emotional, empathy. As previously proposed [64], individuals may feel remorse after perpetrating violent acts. The lack of prosocial behaviour and empathy and proneness to violence could be explained by the history of childhood abuse and/or rejection [20]. Our results confirm this argument, as a larger history of childhood rejection was associated to poor cognitive empathy, especially in HA IPV perpetrators.

A previous study revealed that those IPV perpetrators with higher alcohol consumption presented worse WCST performance than those without alcohol consumption but who were smokers [30]. Moreover, we have previously stated that IPV perpetrators present lower cognitive flexibility than non-violent men [33]. Our current data revealed that HA presented lower cognitive flexibility than the LA IPV perpetrators. Moreover, that low socio-cognitive skills would be related to the maintenance of sexist stereotypes [33] and low social adequacy [65], and may be explained by a previous history of childhood abuse and/or rejection [20]. Our current data confirm this hypothesis, because a larger history of childhood parental rejection, particularly in HA consumers, was associated with high hostile sexism, which was mediated by low cognitive flexibility.

When impulsivity was presented with high anger traits the likelihood of committing domestic violence increases, at least in the case of women [66]. Moreover, in healthy men volunteers high impulsivity traits [31] and anger traits [32] were related to aggressive behavior after alcohol consumption. Our data support those results because HA consumption IPV perpetrators presented higher impulsivity and anger traits and expression. Moreover, a masculinized 2D:4D ratio has been related with high sensation seeking [24], physical aggression [25] in healthy men and as a marker of alcohol dependence [22,23]. Hence, those traits would be explained by an early prenatal androgen exposure. We have observed, especially in HA IPV perpetrators, that a masculinized right 2D:4D ratio was associated to high anger expression, which was mediated by high impulsivity traits. On the other hand, a masculinized brain is characterized by poor empathic skills [58]. In this sense, our results support that a masculinized right 2D:4D ratio was associated to poor cognitive empathy, which was in turn mediated by poor emotional decoding process.

A limitation of our study is the cross-sectional and non-experimental design which does not allow causality to be addressed. Another limitation included was that the results are based specifically in men. Nevertheless, women were excluded. Hence, this fact potentially weakens the external validity. Additionally, the absence of information about the IPV perpetrators’ current severity of alcohol dependence (ages of dependence, alcohol consumption per day and poly drug use) limited the externalizing value of our study. For this reason, future work might replicate these findings contemplating those alcohol consumption factors and two additional groups with and without alcohol consumption, but both without aggressive behavior or domestic violence. Moreover, it would be necessary consider a women sample.

## 5. Conclusions

Parental rejection during childhood and early androgen exposure are relevant factors for the development of violence and the lack of adequate empathy in adulthood. Thus, maltreated and masculinized men present impairments in their socio-cognitive skills, which may make them more prone to violence. Moreover, alcohol abuse may act as a catalytic factor in this relationship as major deficits related to high alcohol consumption involve violent resolution tactics, high sexist stereotypes and larger risk of recidivism. The results of this research could benefit rehabilitation programs designed for violent abusers that employ a communitarian and psychotherapeutic perspective focused primarily on changing beliefs, biases, and/or cognitive distortions of offenders. This study joined with our previous research findings [33,67] also contribute to the creation of new coadjutant of intervention programs, focused on the rehabilitation of basic executive functions and emotional recognition skills training techniques. An emphasis on emotional intelligence training may be useful to increase the accuracy in emotional face processing by means of correctly contextualizing and accuracy of them. Furthermore, it would be essential to treat alcohol dependence as it plays a key role in the development of the socio-cognitive impairments. Moreover, the alcohol intake increase the proneness to violence and its recidivism by restricting the perception of external and internal information, the focusing of conscious perception on a small number of salient stimuli to neglect some information increases the likelihood of a violent reaction.
